# Prevalence of proximal contact loss between implant-supported fixed prosthesis and adjacent teeth and associated factors: A systematic review and meta-analysis

**DOI:** 10.34172/japid.2022.023

**Published:** 2022-11-12

**Authors:** Shima Ghasemi, Laleh Oveisi-Oskouei, Ali Torab, Hanieh Salehi-Pourmehr, Amirreza Babaloo, Nafiseh Vahed, Nasrin Abolhasanpour, Sina Taghilou, Atieh Ghasemi

**Affiliations:** ^1^Department of Prosthodontics, Faculty of Dentistry, Tabriz University of Medical Sciences, Tabriz, Iran; ^2^Student Research Committee, Tabriz University of Medical Sciences, Tabriz, Iran; ^3^Research Center for Evidence-based Medicine, Iranian EBM Center: A Joanna Briggs Institute Center of Excellence, Tabriz University of Medical Sciences, Tabriz, Iran; ^4^Department of Periodontology and Implant Dentistry, Faculty of Dentistry, Tabriz University of Medical Sciences, Tabriz, Iran; ^5^Department of Pediatrics, Faculty of Dentistry, Tabriz University of Medical Sciences, Tabriz, Iran

**Keywords:** Adjacent teeth, prosthesis, proximal contact loss, systematic review

## Abstract

**Background.** This systematic review and meta-analysis investigated the prevalence of proximal contact loss and its associated factors.

**Methods.** A bibliographic search was conducted in June 2021 with no limitation in the article date or language and updated in January 2022 by hand searching. There was no time limit on the search to retrieve all studies. The search included randomized controlled trials or quasi-experiments, and cross-sectional or cohort studies were included in the absence of these studies. Two authors screened the title and abstract. After evaluating the full texts of selected articles, irrelevant studies and or non-English papers that were impossible to translate were excluded. Disagreements between the re­viewers’ selection process were resolved by debate on the eligibility of studies. Standardized critical appraisal instruments from the Joanna Briggs Institute for different types of studies were used to assess the studies’ quality. Comprehensive Meta-Analysis (CMA) software (Version 2.2; Biostat, Englewood, NJ) was used for data analysis.

**Results.** The proximal contact loss (PCL) frequency was %29. According to the results, the frequencies of PCL for the distal and mesial aspects were %7 and %21, respectively. The meta-analysis results showed that the contact loss events on the mesial aspect were statistically higher than on the distal aspect (P<0.0001). There were no significant differences between other associated factors such as the mandibular or maxillary arch, retention type, opposing dentition, implant type, molar or non-molar, parafunction behaviors, and vitality of adjacent teeth. There was a significant association between bone loss and PCL, and in individuals with bone loss >%50, the proximal contact loss was higher (OR: %95[ 2.43 CI: 4.03‒1.47], P=0.0006). The PCL in the anterior area was lower than in the posterior area (P=0.004). Although the frequency of contact loss in females was higher than in males, this rate was not statistically significant.

**Conclusion.** The PCL on the mesial aspect and the posterior area was high. In individuals with bone loss >%50, the proximal contact loss was higher than in others.

## Introduction

 Implant-supported restorations are highly successful and are among the current standard of care for restoring and replacing lost teeth.^1,2^Although this treatment is considered reliable, providing comfort by simulating the appearance, high survival rates, and function of natural teeth,^3,4^ implant therapy entails a risk of biological (peri-implant mucositis or peri-implantitis) and mechanical (screw loosening, screw fracture, or fracture of the superstructure material) complications with individual variabilities in prosthetic designs.^5,6^

 Proximal contact loss (PLC) is one of the most important postoperative complications. However, Gibbard and Zarb^7^ (2002) described the absence of a proximal contact point as the loss of the contact point between implant-supported fixed prostheses and the adjacent tooth for the first time. After dental implant placement, interproximal contact loss is a prosthetic complication with a high prevalence of 18–66% in the maxilla and 37–54% in the mandible three months after prosthetic treatment.^8^

 There is a consensus that modifying the spatial relationship between an implant and the supporting bone is impossible, even in cases that have undergone significant changes because of craniofacial growth.^9^ In addition, subjects that exhausted their growth potential may experience tooth movement,^10^ generally observed in mandibular incisor crowding, the overeruption of maxillary incisors, and mesial drift that usually occurs in mandibular first molars.^11^ The possibility of mesial tipping or drifting of adjacent teeth may cause a significant opening in the mesial contact point.^12^ Biofunctional aspects could be effective in altering tooth positions so that changes in the chewing pattern or the contact points lead to alterations in proximal contacts.^13^ PCL affects periodontal health, and loss of the contact points between a tooth-supported fixed restoration and the adjacent teeth is associated with food impaction.^14^

 Thus, this systematic review and meta-analysis investigated the prevalence of proximal contact loss and its relevant factors and effects on periodontal/pre-implant tissue conditions in the embrasures between implants and adjacent teeth that affect the health of adjacent teeth and may cause dental caries, periodontal problems, and mucositis

## Methods

###  Search strategy

 A bibliographic search was conducted in Web of Sciences, PubMed, Scopus, ProQuest, Embase, Medline (via Ovid), Google Scholar, Cochrane Library, ongoing trials registers, and conference proceedings in June 2021, with no limitation in the article date or language, and updated at January 2022 by hand searching. For this purpose, the following keywords were searched: “dental implant,” “dental prosthesis,” “implant-supported prosthesis,” “FDP,” “tooth migration,” “adjacent teeth,” “proximal contact loss,” “open contacts,” “contact tightness,” and “food impaction.” In addition to the strategic search, a manual search was carried out in the references of related articles to reduce the possibility of missing studies.

###  Inclusion and exclusion criteria

 Inclusion criteria were as follow: studies analyzing the reasons for PCL between the implant-supported fixed prosthesis and adjacent teeth in individuals having lost a tooth for any reason after 18 years of age with sufficient bone remaining, who were candidates for dental implant treatment. There was no time limit on the search to retrieve all studies. The search included randomized controlled trials or quasi-experiments. In the absence of these studies, cross-sectional or cohort studies were also included. Preliminary articles were selected and reviewed based on inclusion criteria. Two authors screened the titles and abstracts. After evaluating the full texts of the selected articles, irrelevant studies or non-English papers that were impossible to translate were excluded. Disagreements between the reviewers’ selection processes were resolved by discussing the eligibility of studies.

###  Assessment of methodological quality

 For this purpose, two independent reviewers assessed the eligible studies for critical appraisal according to standardized critical appraisal instruments from the Joanna Briggs Institute for different types of studies, including cohort, RCT, and quasi-experimental studies.^15^ Any disagreements between the two reviewers were resolved by discussion or consultation with the third reviewer. Studies with a ½ or higher score level in questions were included as high- or moderate-quality studies (Table 1).

**Table 1:** Critical appraisal results of eligible studies

**Study**	**Q1**	**Q2**	**Q3**	**Q4**	**Q5**	**Q6**	**Q7**	**Q8**	**Q9**
**Gohil et al (1973)**	Yes	UC	No	Yes	NA	Yes	Yes	Yes	Yes
**Gibbard and Zarb (2002)**	No	Yes	No	No	NA	No	UC	UC	No
**Wei et al (2008)**	Yes	UC	NA	Yes	Yes	Yes	Yes	Yes	Yes
**Koori et al (2010)**	Yes	Yes	UC	Yes	NA	Yes	Yes	Yes	Yes
**Ahmad (2011)**	NA	Yes	Yes	Yes	NA	Yes	Yes	Yes	Yes
**Byun et al. (2015)**	Yes	Yes	No	Yes	NA	Yes	Yes	Yes	Yes
**Ren et al. (2016)**	No	Yes	No	Yes	Yes	Yes	UC	Yes	No
**Wong et al. (2015)**	No	UC	No	Yes	NA	Yes	Yes	Yes	Yes
**Akhtar et al (2015)**	Yes	NA	UC	Yes	Yes	Yes	Yes	UC	Yes
**Varthis et al. (2016)**	No	Yes	No	Yes	NA	UC	No	UC	Yes
**Pang et al. (2017)**	Yes	Yes	No	Yes	Yes	Yes	Yes	Yes	Yes
**French et al. (2019)**	Yes	Yes	Yes	Yes	NA	UC	UC	Yes	Yes
**Shi et al. (2019)**	No	Yes	No	Yes	Yes	Yes	Yes	Yes	Yes
**Jo et al (2019)**	Yes	Yes	NA	Yes	Yes	Yes	Yes	Yes	UC
**Almalki et al (2019)**	Yes	Yes	UC	Yes	Yes	Yes	Yes	Yes	UC
**Bompolaki et al. (2020)**	No	Yes	No	Yes	NA	Yes	Yes	Yes	Yes
**Kandathilparambil et al. (2020)**	No	No	No	UC	Yes	No	Yes	UC	NA
**Liang et al. (2020)**	No	Yes	Yes	Yes	NA	Yes	Yes	Yes	Yes
**Saber et al. (2020)**	Yes	Yes	No	Yes	UC	UC	UC	Yes	Yes
**Yen et al. (2020)**	Yes	Yes	No	Yes	NA	UC	Yes	Yes	Yes
**Wang et al. (2020)**	Yes	Yes	Yes	Yes	NA	UC	UC	No	No
**Mehanna et al (2021)**	UC	Yes	NA	Yes	Yes	Yes	Yes	Yes	Yes
**Latimer et al. (2021)**	Yes	Yes	UC	Yes	Yes	Yes	Yes	Yes	NA
**Chen et al. (2021)**	UC	No	NA	Yes	Yes	Yes	Yes	Yes	Yes

NA: not applicable, UC: unclear

###  Data extraction

 A standardized data extraction form was used to record the relevant information of selected studies as follows: author(s), year of publication, design of the study, number of patients, age, number of implants, number of implant prostheses, retention type, number of patients with systemic illness, number of patients with periodontal disease, number of patients with parafunctional habits, number of smokers, bone level, traumatic occlusion (plunger cusp), vitality of adjacent teeth, root configuration of adjacent teeth, distribution of prostheses, number of proximal contacts, number of PCL, opposing dentition, follow-up years, assessment, and conclusion (supplementary file).

###  Statistical analysis

 Quantitative papers, whenever possible, were pooled in the statistical meta-analysis using the JBI-MAStARI and Comprehensive Meta-Analysis (CMA) software (version 2.2; Biostat, Englewood, NJ). All the results were subject to double data entry. Heterogeneity was assessed statistically using the standard chi-squared test and explored using subgroup analysis based on the different quantitative study designs included in this review. Where statistical pooling was not possible, the findings were presented in a narrative form, including tables and figures. Q statistic was used for detecting heterogeneity within the studies. In addition, I^2^ statistic was applied to estimate the effect of heterogeneity in the studies. I^2^ was considered low at 75%. A fixed-effect model was applied in cases with no statistical difference in heterogeneity (P≥0.05); otherwise, a random-effect model was used. Furthermore, funnel plots were used to assess the publication bias.

## Result

**Figure 1:**
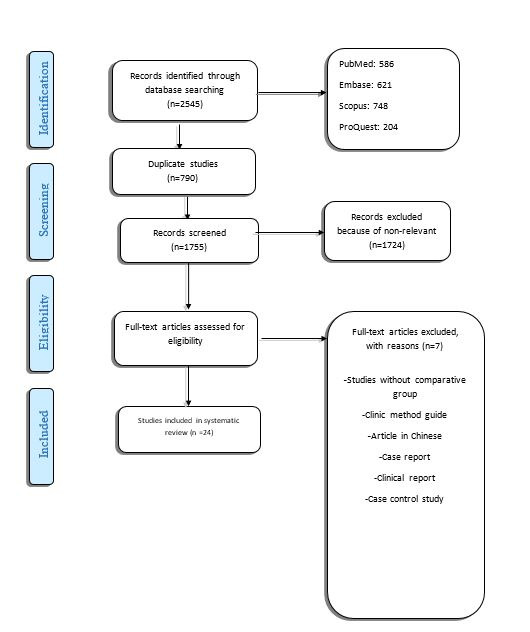
Search results and study selection and inclusion process.

 The details of publications during selection and elimination are summarized in the Preferred Reporting Items for Systematic Reviews and Meta-analysis protocols (PRISMA) study flow diagram (Figure 1).

 Of 2545 records identified through database^16^ searching, 24 articles had selection criteria for full-text reading. Nine full-text studies did not meet the inclusion criteria and were excluded. Of the 19 included articles, one article was added to this study by manual search.^7^ Of included studies^(7,16-38)^ 14 had a retrospective design,^(17-19,21,22,24-28,30,32,37,38)^ 7 studies^(7,20,23,29,33,35,36)^ were prospective, and 3 were cross-sectional studies.^(16,31,34)^ The details and data extracted from each study are summarized in the supplementary file.

 Of 7480 implants, 570 implants were placed in the maxilla and 638 in the mandible. Moreover, while 7 articles^17,19,20,23,27,29,31^included research that was limited to posterior implant-supported prostheses, 3 study^8,16,34^ reported both anterior and posterior implant-supported prostheses, and only one article^26^ included anterior prostheses.

###  Meta-analysis results

**Figure 2:**
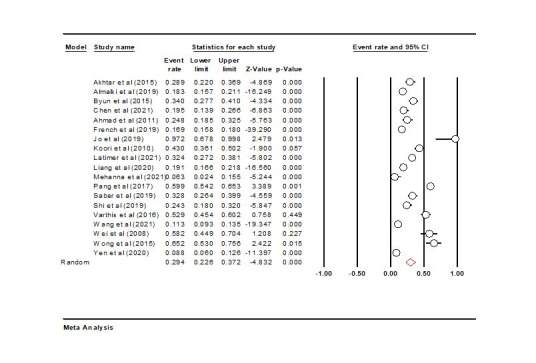
The frequencies of proximal contact loss.

 Nineteen studies were eligible for meta-analysis. According to the results of the meta-analysis, the frequency of PCL was 29.4% (95% CI: 22.6‒37.2%) (Q-value=643.491, I^2^=97.20) (Figure 2). However, I^2^ was >50%, indicating the high heterogeneity of the studies. We, therefore, used the random-effect model here.

**Figure 3:**
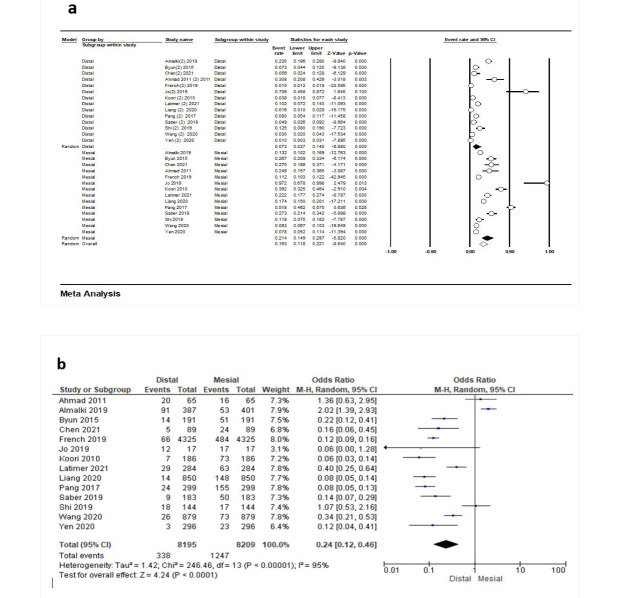
a. Frequency of PCL in terms of mesial and distal aspects. b. Comparison of the PCL in terms of mesial or distal aspects.

 Fourteen studies^16,18,23-30,32-34,37^were eligible to evaluate the frequency of PCL based on mesial or distal aspects. According to the results, the frequency for the distal aspect was 7.3% (95% CI: 3.7‒14.0%, Q-value=465.431, df=13, P<0.001, I^2^=97.20). Concerning the mesial aspect, this rate was 21.4% (95% CI: 14.9‒29.7%, Q-value=484.197, df=14, P<0.001, I^2^=97.31). The overall point estimate was 16.3% (95% CI: 11.8‒22.1%, Q-value=1155.458, df=27, P<0.001, I^2^=97.66) (Figure 3a, 3b). Also, the meta-analysis showed that the contact loss event on the mesial aspect was statistically higher than on the distal aspect (P<0.0001).

**Figure 4:**
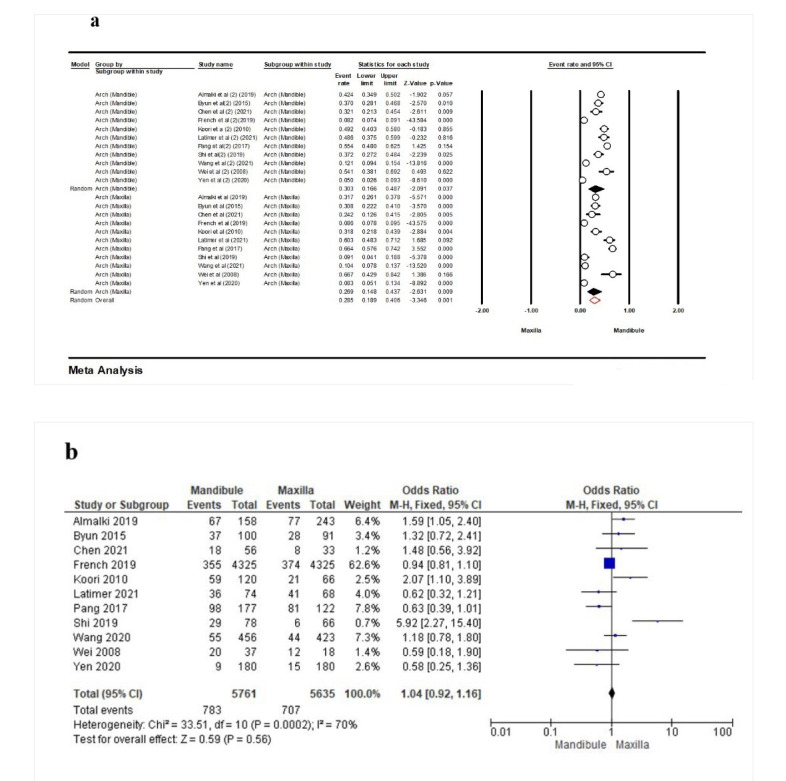
a. Frequency of contact loss in mandible over maxillary arch, b. Comparison of the results of proximal contact loss based on the arch

 Eleven studies^16,23,26,28-30,32-34,37,38^were eligible to evaluate the frequency of PCL in terms of the mandibular or maxillary arch. According to the results, this frequency for the mandible was 30.3% (95% CI: 16.6‒48.7%, Q-value=649.789, df=10, P<0.001, I^2^=98.46), with 26.9% (95% CI: 14.8‒43.7%, Q-value=482.731, df=10, P<0.001, I^2^=97.92) for the maxilla. Overall, this rate was 28.5% (95% CI: 18.9‒40.6%, Q-value=1141.9111, df=21, P<0.001, I^2^=98.16) (Figure 4a). Also, the results of the meta-analysis showed that the contact loss event in the mandibular or maxillary arch was similar (OR: 1.04 [95% CI: 0.92‒1.16], P=0.56) (Figure 4b).

**Figure 5:**
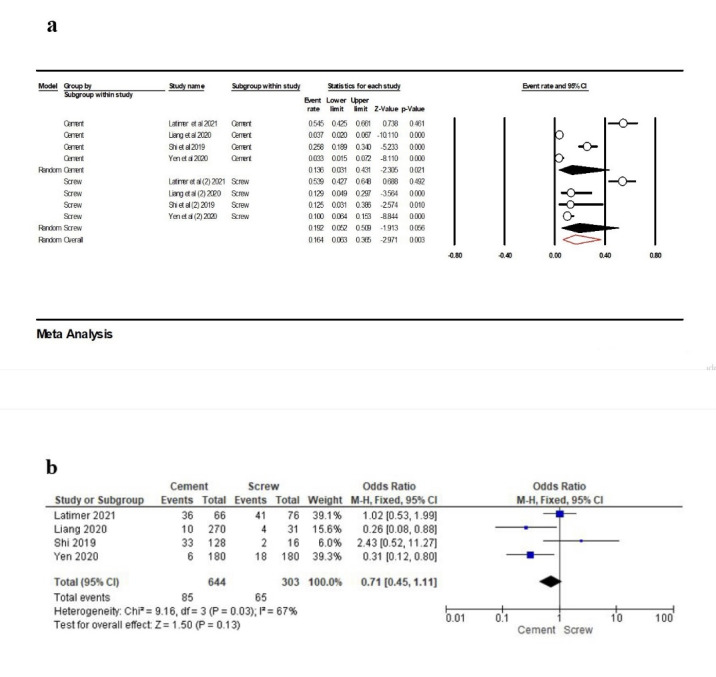
a. Frequency of contact loss in terms of retention type, b. Comparison of the results of proximal contact loss based on the retention type.

 Four studies^24,29,32,34^were included in the meta-analysis to compare the results of PCL in terms of the retention type. The point estimate for cement retention was 13.6% (95% CI: 3.1‒43.1%, Q-value=99.099, df=3, P<0.001, I^2^=96.97), and for screw retention this rate was 19.2% (95% CI: 5.2‒50.9%, Q-value=52.860, df=3, P<0.001, I^2^=94.32). The overall point estimate was 16.4% (95% CI: 6.3‒36.5%, Q-value=151.465, df=7, P<0.001, I^2^=95.43) (Figure 5a). According to the meta-analysis, there was no association between the retention type and PCL (OR: 0.71 [95% CI, 0.45‒1.11], P=0.13) (Figure 5b).

**Figure 6:**
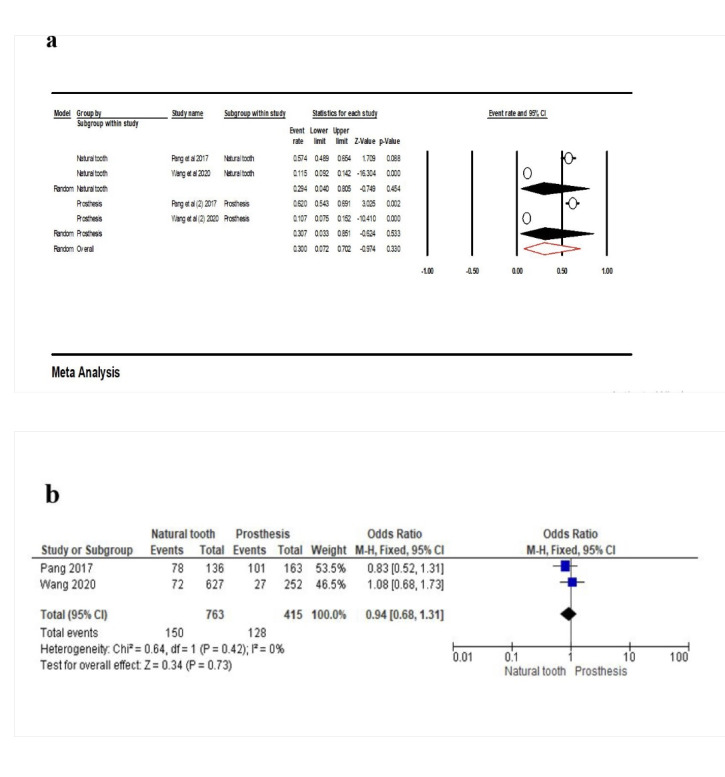
a. Frequency of contact loss in terms of Opposing Dentition, b. Comparison of the results of proximal contact loss based on the opposing dentition.

 Two studies^23,30^were included in the meta-analysis to investigate the effect of opposing dentition on PCL. According to the results, the point estimate for natural tooth was 29.4% (95% CI: 4‒80.5%, Q-value=119.532, df=1, P<0.001, I^2^=99.16). This rate for prosthesis was 30.7% (95% CI: 3.3‒85.1%, Q-value=100.768, df=1, P<0.001, I^2^=99.01). The overall point estimate was 30% (95% CI: 7.2‒70.2%, Q-value=220.300, df=3, P<0.001, I^2^=98.75) (Figure 6a). According to the meta-analysis, there was no association between the opposing dentition and PCL (OR: 0.94 [95% CI, 0.68‒1.31], P=0.73) (Figure 6b).

**Figure 7:**
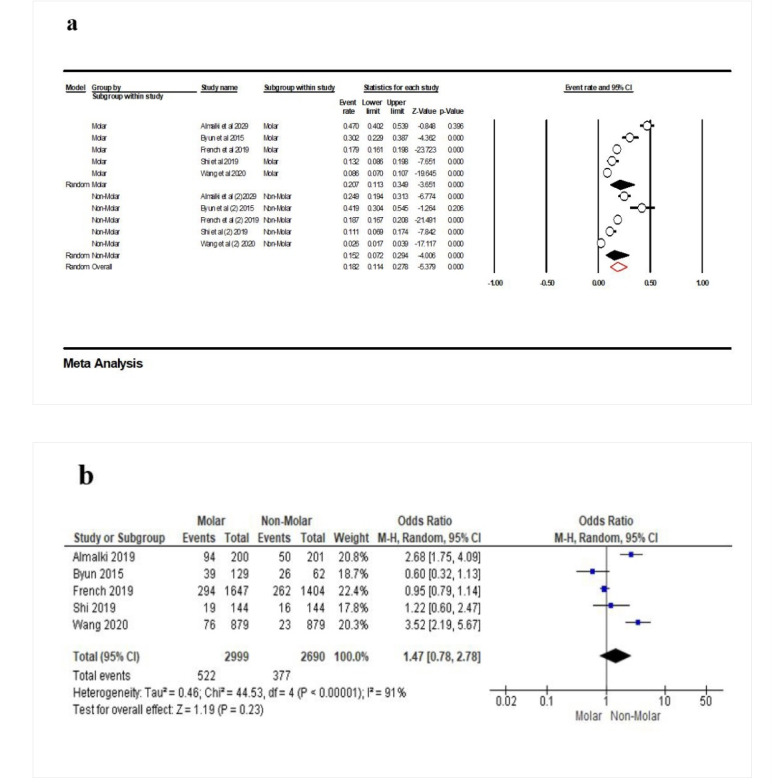
a. Frequencies of contact loss in terms of molar or non-molar teeth. b. Comparison of the results of proximal contact loss in the molar or non-molar teeth.

 According to five studies^16,26,28,29,30^included in the meta-analysis, the frequency of contact loss for molars was 20.7% (95% CI: 11.3‒34.9%, Q-value=159.989, df=4, P<0.001, I^2^=97.50), and for non-molars, the point estimate was 15.2% (95% CI: 7.2‒29.4%, Q-value=131.007, df=1, P<0.001, I^2^=96.94), with 18.2% overall (95% CI: 11.4‒27.8%, Q-value=290.996, df=8, P<0.001, I^2^=96.92) (Figure 7a). According to the meta-analysis, there was no association between the molar or non-molar teeth and PCL (OR: 1.47 [95% CI, 0.78-2. 78], P=0.23) (Figure 7b).

**Figure 8:**
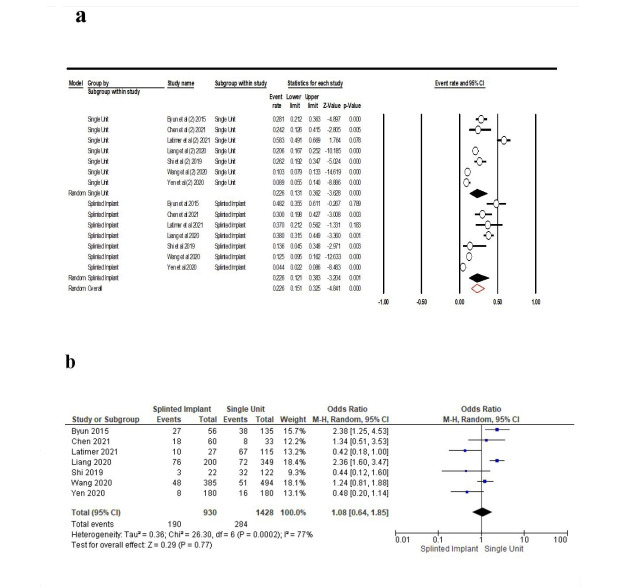
a. Frequencies of contact loss in terms of the splinted implant and non-splinted (single unit) implants. b. Comparison of the results of proximal contact loss in the splinted implant or single units.

 Seven studies^16,24,29,30,31,32,34^were included in the meta-analysis to evaluate the frequency of contact loss in terms of splinted and non-splinted (single unit) implants. The results showed a similar point estimate for both splinted and non-splinted implants, with 22.6% (95% CI: 12.1‒38.3%, Q-value=129.352, df=6, P<0.001, I^2^=95.36) for splinted and single-unit implants (point estimate: 22.6% [95% CI: 13.1‒36.2%, Q-value=96.467, df=6, P<0.001, I^2^=93.78]). Overall this rate was 22.6% (15.1%-32.5%, Q-value=225.819, df=12, P<0.001, I^2^=94.28) (Figure 8a). According to the results of the meta-analysis, there was no association between the type of implant and PCL (OR: 1.08 [95% CI, 0.64-1. 14), P=0.77]) (Figure 8b).

**Figure 9:**
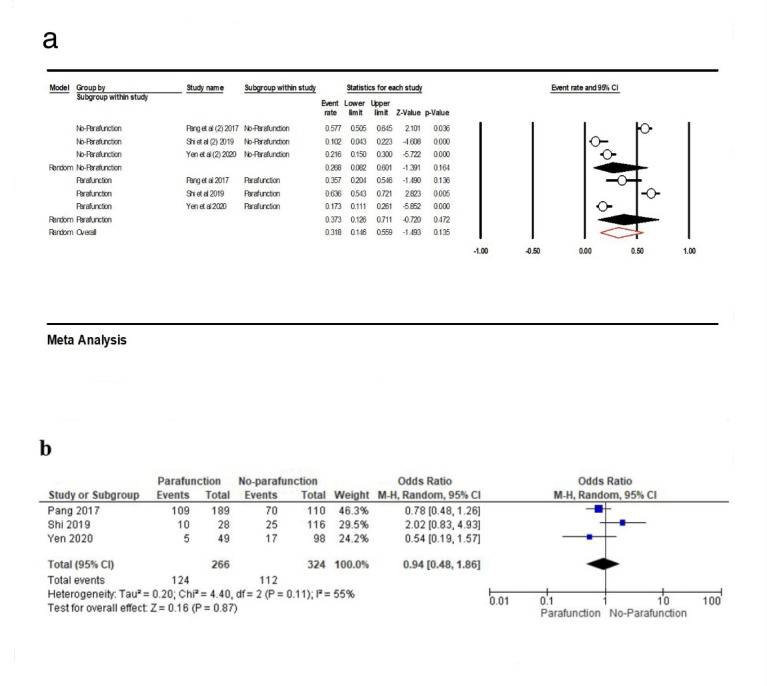
a. Frequencies of contact loss in terms of the parafunctional and non-parafunctional habits. b. Comparison of the results of proximal contact loss in terms of the function.

 The point estimate of three included studies^23,29,32^for parafunction habits was 37.3% (95% CI: 12.6‒71.1%, Q-value=41.577, df=2, P<0.001, I^2^=95.19), and for non-parafunction, it was 26.8% (95% CI: 8.2‒60.1%, Q-value=52.208, df=2, P<0.001, I^2^=96.16), with overall 31.8% (14.6‒55.9%, Q-value=93.846, df=5, P<0.001, I^2^=94.67) (Figure 9a). According to the results of the meta-analysis, there was no association between the parafunctional habits and PCL (OR: 0.94 [95% CI, 0.48‒1.86], P=0.87) (Figure 9b).

**Figure 10:**
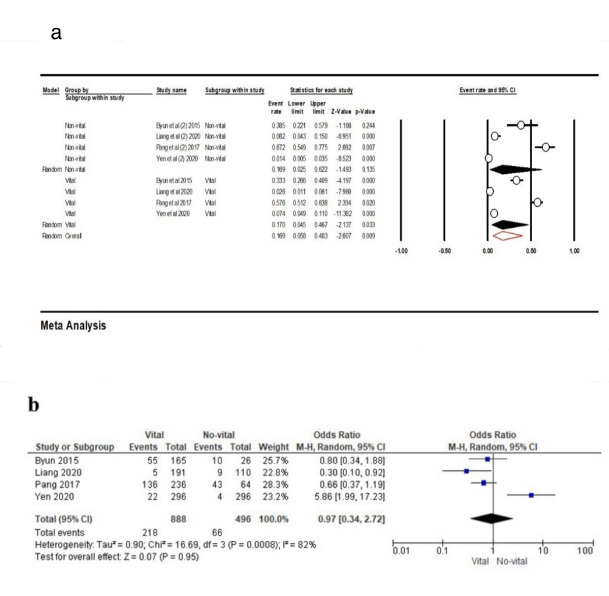
a. Frequencies of contact loss in terms of the vitality of adjacent teeth. b. Comparison of the results of proximal contact loss in terms of the vitality of adjacent teeth.

 To investigate the frequency of contact loss in terms of the vitality of adjacent teeth, Four studies^16,23,24,32^ were included for this outcome; the results showed that the frequency of contact loss in non-vital adjacent teeth was 16.9% (95% CI: 2.5‒62.2%, Q-value=102.407, df=3, P<0.001, I^2^=97.07), and for vital adjacent teeth this rate was similar, i.e., 17% (95% CI: 4.5‒46.7%, Q-value=167.107, df=3, P<0.001, I^2^=98.20). Overall, the point estimate was 16.9% (95% CI: 5.8‒40.3%, Q-value=271.416, df=8, P<0.001, I^2^=97.42) (Figure 10a). According to the results of the meta-analysis, there was no association between the vitality of adjacent teeth and PCL (OR: 0.97 [95% CI, 0.34‒2.72], P=0.95) (Figure 10b).

**Figure 11:**
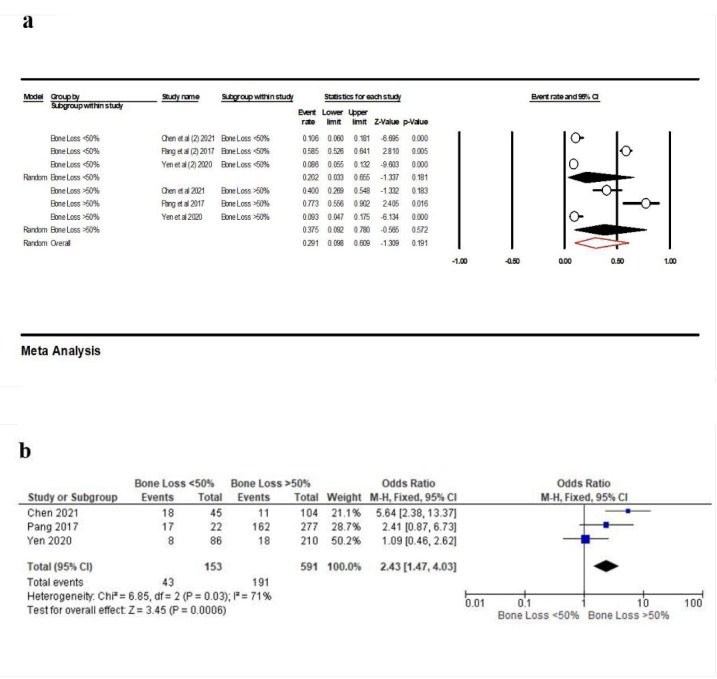
a. Frequencies of contact loss in terms of the amount of bone loss. b. Comparison of the results of proximal contact loss in terms of bone loss.

 For evaluating the association of bone loss with PCL, we categorized the results based on the amount of bone loss: <50% or >50%, and three studies (23, 32, 33) were eligible for meta-analysis. When bone loss was <50%, the frequency of contact loss was 20.2% (95% CI: 3.3‒65.5%, Q-value=130.375, df=2, P<0.001, I^2^=98.46), and in the cases with bone loss >50%, the point estimate was 37.5% (95% CI: 9.2‒78.0%, Q-value=33.255, df=2, P<0.001, I^2^=93.98). Overall, this rate was 29.1% (95% CI: 9.8‒60.9%, Q-value=165.724, df=5, P<0.001, I^2^=96.98) (Figure 11a). According to the results of the meta-analysis, there was a significant association between bone loss and PCL, and in individuals with bone loss >50%, the proximal contact loss was higher (OR: 2.43 [95% CI, 1.47‒4. 03], P=0.0006) (Figure 11b).

**Figure 12:**
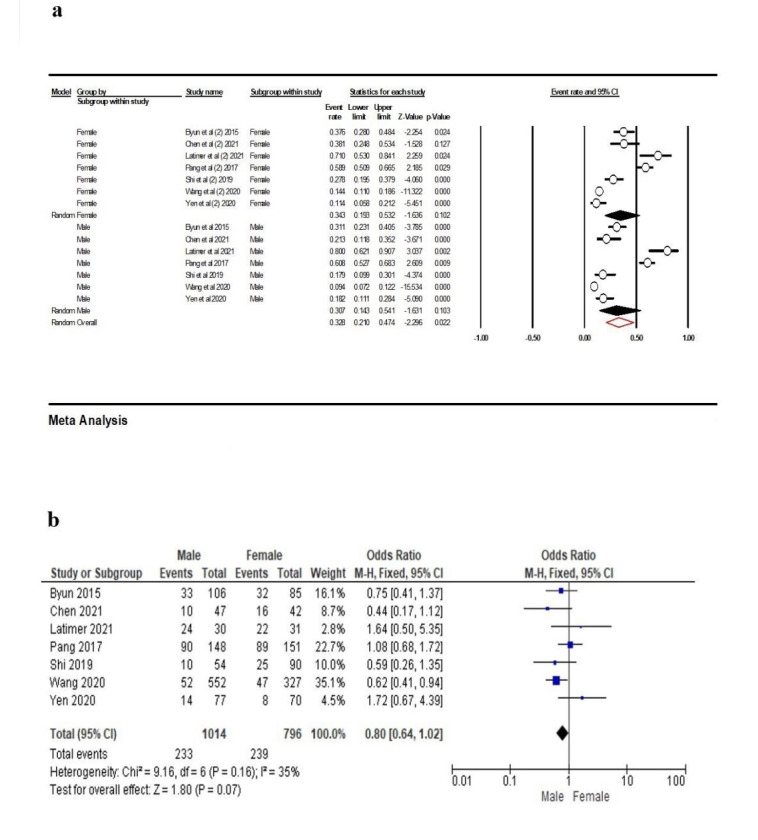
a. Frequencies of contact loss in terms of gender. b. Comparison of the results of proximal contact loss in both genders.

**Figure 13:**
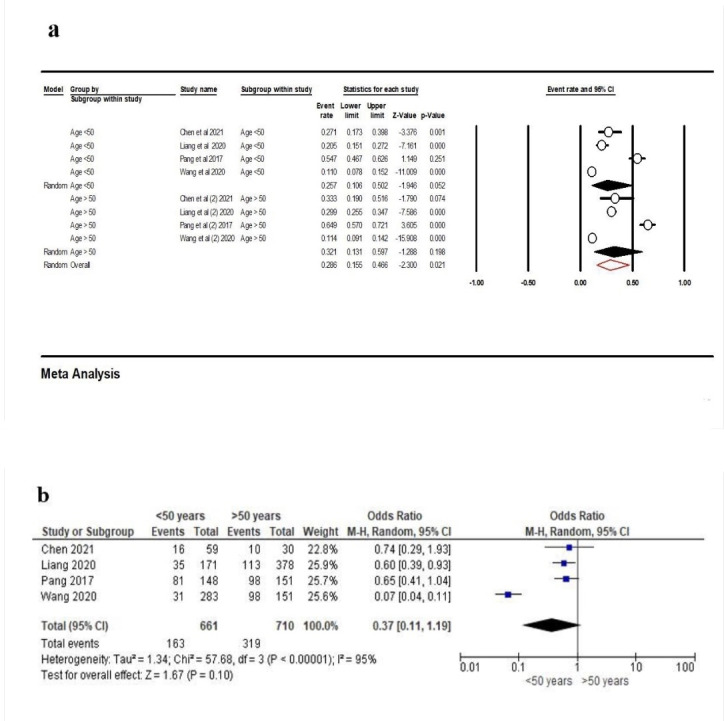
a. Frequencies of contact loss in terms of the age. b. Comparison of the results of proximal contact loss in elderly individuals.

 In the seven studies (16, 23, 29, 30, 32-34) included in meta-analysis for gender differences, the results showed that in females the frequency of contact loss was 32.7% (95% CI: 29.2‒36.5%, Q-value=119.956, df=6, P<0.001, I^2^=94.99), with 26.2% (95% CI: 23.1‒29.5%, Q-value=119.956, df=6, P<0.001, I^2^=96.70) in males, and overall, 32.8% (95% CI: 21.0‒47.4%, Q-value=309.956, df=13, P<0.001, I^2^=95.79) (Figure 12a). Also, according to the results presented in Figure 13, the total event rate of contact loss in females was higher than in males. However, this rate was not statistically significant (OR: 0.80 [95% CI, 0.67‒4.39], P=0.07) (Figure 12b).

**Figure 14:**
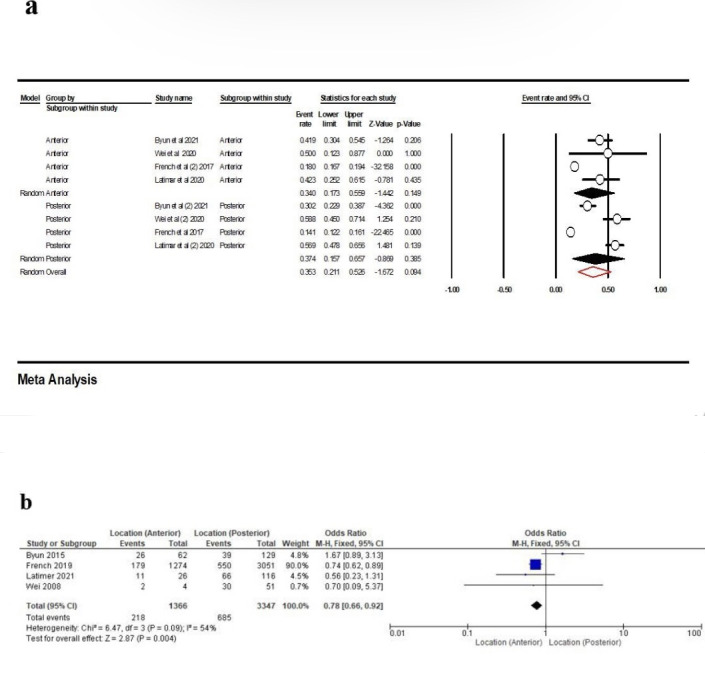
a. Frequencies of contact loss in terms of location. b. Comparison of the results of proximal contact loss in terms of location.

 Four studies^23,24,30,33^were eligible for evaluating PCL in terms of age category. According to the results, the frequency of contact loss in patients <50 years of age was 25.7% (95% CI: 10.6‒50.2%, Q-value=88.463, df=3, P<0.001, I^2^=96.60); for individuals >50 years of age, it was 32.1% (95% CI: 13.1‒59.7%, Q-value=158.196, df=3, P<0.001, I^2^=98.10), and overall, 28.6%, (95% CI: 15.5‒46.6%, Q-value=246.659, df=7, P<0.001, I^2^=97.16) (Figure 13a). Also, according to the results presented in Figure 14, the total event rate of contact loss in individuals <50 years of age was lower than in patients >50 years of age. However, this rate was not statistically significant (OR: 0.37 [95% CI, 0.11‒1.19], P=0.10) (Figure 13b).

 Based on the four studies,^16,26,34,38^ which were included in the meta-analysis, the contact loss rate in the anterior area was 34% (95% CI: 17.3‒55.9%, Q-value=31.293, df=3, P<0.001, I^2^=90.41), with 37.4% (95% CI: 15.7‒65.7%, Q-value=150.752, df=3, P<0.001, I^2^=98.01) in the posterior area, and overall, 35.3% (95% CI: 21.1‒52.6%, Q-value=185.637, df=7, P<0.001, I^2^=96.22) (Figure 14a). According to our results, there was a significant correlation between the location and the PCL, and in the anterior area, it was lower than the posterior area(OR: 0.78 [95% CI, 0.66‒0. 92], P=0.004) (Figure 14).

###  Publication bias

**Figure 15:**
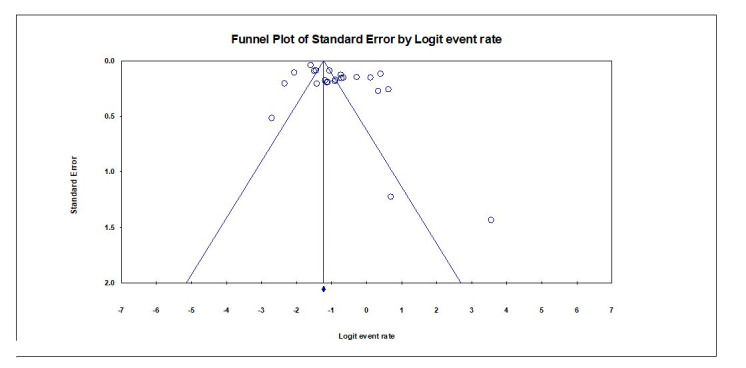
Publication bias of the included studies.

 There was no evidence of publication bias in favor of studies reporting a high frequency of PCL using the Begg-Mazumdar test (Tau=0.0065, z-value=0.0378, two-tailed P=0.969) (Figure 15).

## Discussion

 The proximal contact loss (PCL) frequency was 29%. According to the results, the frequency of PCL for the distal aspect was 7%, with 21% for the mesial aspect. The meta-analysis results showed that the contact loss event on the mesial aspect was statistically higher than on the distal aspect (P<0.0001). There was no significant difference between other associated factors such as the arch of the mandible or maxilla, retention type, opposing dentition, implant type, molar or non-molar teeth, parafunctional habits, and vitality of adjacent teeth. There was a significant association between bone loss and PCL, and in individuals with bone loss >50%, the proximal contact loss was higher (OR: 2.43 [95% CI, 1.47‒4. 03], P=0.0006). The PCL of the anterior area was lower than the posterior area (P=0.004). Although the frequency of contact loss was higher in females than in males, the difference was not significant.

 After the occlusal forces are applied anteriorly for a long time, the adjacent teeth tend to migrate in the mesial direction.^39^ Therefore, the possibility of open proximal contact on the mesial aspect is twice greater than on the distal aspect of implant-supported prostheses. A continuous increase in the interproximal gap was observed, with a three-fold tighter contact in the distal than in the mesial contact.^20^ Mesial migration happens in a 3D complex pattern with labial or lingual components following an adaptive reaction to the continuous occlusal forces and supporting structure growth.^27,38^ Our findings showed that the frequency of mesial open contact (21.2%) was greater than distal contact (7.7%). The potential factors influencing the PCL at the mesial aspect are mesial migration and traumatic occlusion, such as a plunger cusp, which was reported by only one study (P=0.0046).^24^

 Previous studies have shown a dynamic relationship between interproximal contacts and the occlusal function.^13^ In the relax and rest condition, mandibular proximal contacts were stronger than the maxilla. In contrast, increased maxillary contacts’ strength was observed during the clench condition compared to mandibular contacts.^40^ Overall, there is no significant difference between the strength of mandibular contacts at rest and during clenching.^40^ Therefore, it is evident that occlusal function affects more maxillary contacts than mandibular contacts.^41^ This study demonstrated that PCL is more common in the mandible (27.1%) than in the maxilla (24.5%). However, the difference between the two meta-analyzed proportions was remarkably lower than reported in several studies. This difference may have been due to a higher degree of mesial drift in the mandible.

 Older people may show a decline in resistance to forces by reducing the level of bone located around their teeth.^23^ Some previous studies reported a higher rate of open proximal contacts in aged individuals than in young persons.^37^ Also, an inverse relationship was observed between open proximal contact and bone level around the adjacent teeth.^23^ This study reported a significant association between bone loss and contact loss; therefore, bone loss of <50% leads to higher PCL. Our analysis showed that the frequency of contact loss in individuals >50 years of age (32.1%) was more than that in those <50 years of age; however, the difference was not significant. The frequency of contact loss in females (32.7%) was higher than in males (26.2%).

 It is possible that splinting increases the resistance of prostheses to dental forces and limits tooth migration.^19,23^ Previous research demonstrated that the rate of proximal contact loss near the implants splinted with fixed dental prostheses (FDP) was 2.5 times higher than that adjacent to the single implant-supported restorations. However, splinting of the implants was not considered a significant factor for PCL.^16^ Our results also showed no association between splinted and non-splinted implants and PCL.

 The opposing dentition has a more critical role in developing OPC due to the dynamic relationship of interproximal contacts.^13^ However, no significant association was reported between OPC and the opposing dentition.^19^ OPC can occur when an implant-supported prosthesis is placed out of occlusion^26^ and without opposing antagonists.^23^ Other variables, such as occlusal forces and parafunctional habits, had no significant effect on OPC.^29^ Our results showed no significant relationship between the opposing dentition and contact loos. The current study reported that contact loss in the anterior area was lower than in the posterior area. However, there was no significant difference in the effect of premolar and molar areas on PCL.

 A strategy for removing the implant-supported prostheses from the patient’s oral cavity is screw retention. Nevertheless, screw retention could impact the induction of force to adjacent teeth due to inconsistency in implant prostheses.^42^ Cement retention could be vital in eliminating a potential source of faults related to any possible misfit of implant abutment.^43^ Our analysis showed that the frequency of contact loss in terms of retention type in cement retention, screw retention, and overall was 13.6%, 19.2%, and 16.4%, respectively. There was no significant association between retention type and PCL.

 Different factors can influence interproximal contacts, such as the various patterns of facial growth,^44^ vitality of adjacent teeth,^37^ and the time of day.^40^ Probably, increased contact tightness is not stable and may decrease after applying orthodontic forces to adjacent teeth.^20^ It is essential to inform patients of the possible development of OPC. The follow-up time is one of the most critical factors for investigating OPC development.^23,26^ OPC developed less than three months after implant-supported prostheses delivery and gradually increased over time.^26,38^ According to our study, the incidence of interproximal contact loss increased over time; the differences between various studies might also be due to the follow-up duration. According to our study, no significant difference was observed between implants’ proximity to nonvital and vital teeth.

 In evaluating the relationship between the angulation of natural teeth with the axis of the implant, studies have reported a much higher prevalence of PCL in single-root adjacent teeth than in multi-rooted adjacent teeth.^16,37^ In our research, only two articles mentioned the root configuration of adjacent teeth. Wong et al^19^reported the effect of angulation (P=0.874); Pang et al^23^ reported that the root configuration of the adjacent teeth was significantly associated with the cumulative PCL rate (P<0.05).

 Several factors are associated with the prevalence and the severity of periodontal diseases, including the number of missing teeth,^45^ oral hygiene,^46^and alveolar bone height.^47^ Smoking is considered a risk factor for periodontal disease.^48^ One study showed a significantly higher frequency of periodontal pockets in smokers than nonsmokers. Subsequently, a significant correlation was demonstrated between smoking and periodontal disease.^49^ In this review, only three studies^25,32,36^evaluated the effect of smoking on proximal contact loss, reporting no significant association between them. Also, patients with systemic diseases like diabetes did not exhibit any significant differences in PCL (P=0.389).^32^

 Parafunctional habits are prevalent among patients visiting dentists, and they pose one of the major dental challenges for dentists. These parafunctional habits have a significant undesirable effect on teeth and dental prostheses.^50^ In our review, three studies evaluated the effects of parafunctional habits on PCLof implant-supported fixed prostheses and did not report significant differences in PCL.^19,23,32^Our meta-analysis showed the point estimates of these three studies as follows: 37.3% for parafunctional habits, 26.8% for non-parafunctional habits, and 31.8% for overall habits, with no association between the PCL and parafunctional habits.

 Concerning the efficacy of occlusal appliances in preventing PCL, they can prevent tooth movements. Therefore, they should be effective as a protective factor. Moreover, these appliances would only be effective in preventing PCL occurring due to tooth movement, and no clear etiology can be identified from the available evidence.^31,35^

## Conclusion

 The proximal contact loss (PCL) frequency was 29%. According to the results, the contact loss event on the mesial aspect was significantly higher than on the distal aspect. There was no significant difference between other associated factors such as gender, mandibular or maxillary arch, retention type, opposing dentition, implant type, molar or non-molar teeth, parafunctional habits, and vitality of the adjacent teeth. However, there was a significant association between bone loss and PCL, and the proximal contact loss was higher in individuals with bone loss >50%. In addition, PCL in the anterior area was lower than in the posterior area.

## Acknowledgments

 None.

## Competing interests

 The authors declare no competing interests.

## Authors’ contributions

 SG designed the study. LOO wrote the initial draft of the manuscript. HSP and NA revised the draft. All authors contributed to the manuscript’s writing and critical revision. All authors read and approved the final version of the manuscript.

## Funding

 This study was not financially supported by any entity.

## Availability of data

 The data from the reported study are available upon request from the corresponding author.

## Ethics approval

 The protocol of the present study was approved by the Ethics Committee of Tabriz University of Medical Sciences under the code IR.TBZMED.REC.1400.311 All the patients signed informed consent forms.
